# Investigating a novel surrogate indicator of adipose accumulation in relation to erectile dysfunction

**DOI:** 10.1186/s12944-024-02118-9

**Published:** 2024-05-13

**Authors:** Chen-Yuan Deng, Xin-Peng Ke, Xu-Guang Guo

**Affiliations:** 1 Department of Clinical Laboratory Medicine, Guangdong Provincial Key Laboratory of Major Obstetric Diseases, Guangdong Provincial Clinical Research Center for Obstetrics and Gynecology, The Third Affiliated Hospital of Guangzhou Medical University, Guangzhou, 510150 China; 2 Department of Clinical Medicine, The Third Clinical School of Guangzhou Medical University, Guangzhou, 511436 China

**Keywords:** Erectile dysfunction, METS-VF, Obesity, NHANES

## Abstract

**Introduction:**

Although previous studies have linked obesity and erectile dysfunction, the novel surrogate indicators of adipose accumulation are more essential and dependable factors to consider. Therefore, the primary objective of the current investigation was to examine and clarify the association between metabolic score for visceral fat (METS-VF) and erectile dysfunction.

**Methods:**

Firstly, multivariate logistic regression analysis, smoothed curve fitting, and threshold effect analysis were employed to investigate the association between METS-VF and erectile dysfunction. Mediation analysis was also performed to evaluate the mediating role of homocysteine and inflammation. After that, subgroup analysis was carried out to examine the stability of the correlation of METS-VF with erectile dysfunction in various population settings. Furthermore, the area under the receiver operating characteristic (ROC) curve and eXtreme Gradient Boosting (XGBoost) algorithm were utilized to assess the capability of identifying METS-VF in comparison to the other four obesity-related indicators in identifying erectile dysfunction.

**Results:**

After adjusting for all confounding factors, METS-VF was strongly and favourablely correlated with erectile dysfunction. With each additional unit rise in METS-VF, the prevalence of erectile dysfunction increased by 141%. A J-shaped relationship between METS-VF and erectile dysfunction was discovered through smoothed curve fitting. Marital status, physical activity, and smoking status can potentially modify this association. This finding of the ROC curve suggests that METS-VF had a powerful identifying capacity for erectile dysfunction (AUC = 0.7351). Homocysteine and inflammation mediated 4.24% and 2.81%, respectively.

**Conclusion:**

The findings of the current investigation suggest that METS-VF can be considered a dependable identifying indicator of erectile dysfunction.

## Introduction

With the rising standards of living, there is an increased emphasis on sexual health. Erectile dysfunction, a common sexual disorder among males, affects not only the physical and mental health of affected individuals but also influences the relationships between couples and daily activities [1]. Alarmingly, the incidence of erectile dysfunction in the U.S. population has escalated to 20 million cases [2]. Notably, the incidence among younger men is steadily increasing [3]. Therefore, addressing erectile dysfunction remains a significant challenge for the well-being of men. Current treatments for erectile dysfunction mostly consist of medication and testosterone replacement therapy. Nevertheless, the effectiveness of these methods varies among sufferers, and the treatment cost may not be accessible to all patients. Additionally, the etiology of erectile dysfunction can be either psychological or organic. More importantly, the pathophysiological mechanisms are intricate and diverse, affecting the vascular endothelium, neurons, endocrine, and other factors [4–6]. Therefore, how to effectively treat erectile dysfunction still needs further research by medical experts. Moreover, the risks associated with erectile dysfunction extend beyond mere sexual health issues and may signal the progression of other medical conditions [7–9]. An analysis of 9,457 men found that erectile dysfunction raises the likelihood of experiencing negative cardiovascular events [7]. Manalo et al. demonstrated a correlation between erectile dysfunction and an increased likelihood of experiencing anxiety and sadness in patients [8]. Mazzilli et al. discovered a high association between the degree of erectile dysfunction and disrupted glucose metabolism by studying 1,332 subjects [9]. Consequently, it is imperative to investigate the risk elements correlated with erectile dysfunction.

The prevalence of obesity has emerged as a significant threat to human well-being, impacting a staggering population of over 2 billion individuals [10]. The body mass index (BMI) is widely employed as a fundamental indicator of obesity in epidemiological studies. Nevertheless, it presents limitations in appropriately evaluating the distribution of adipose tissue throughout the body [11]. Strong evidence indicates that central adiposity serves as a standalone factor that can predict the occurrence of erectile dysfunction in older men [12]. Although waist circumference (WC) is favored by both academics and clinicians due to its simplicity and high accuracy in assessing central obesity, it is crucial to recognize the significant correlation between BMI and WC [13]. Furthermore, research suggests that a high WC is associated with reduced all-cause mortality and heart failure mortality in comparison to those with WC within the usual range [14]. This surprising observation has been termed the “obesity paradox”. Given the established link between cardiovascular disease (CVD) and erectile dysfunction, the existence of the “obesity paradox” in the context of erectile dysfunction warrants further investigation. These findings may limit the use of WC as a standalone metric for assessing central obesity. Therefore, developing novel surrogate indicators of adipose accumulation that are independent of BMI and WC should be an immediate priority. The study conducted by Bello-Chavolla OY et al. introduces an innovative obesity index termed the Metabolic Score for Visceral Fat (METS-VF). In comparison with several other surrogate indicators of adipose accumulation, this marker has demonstrated a superior capacity to assess obesity, particularly visceral obesity and to evaluate cardiometabolic risk effectively [15]. Previous investigations have established a correlation of METS-VF with an increased susceptibility to CVD events and all-cause mortality [16]. Given the shared risk factors between CVD and erectile dysfunction [17], it is plausible to suggest a potential association between METS-VF and erectile dysfunction.

The focus of the investigation intended to examine the possible correlation between METS-VF and erectile dysfunction. In addition, four evaluation indicators of obesity, including WC, waist-to-height ratio (WHtR), lipid accumulation product (LAP), and visceral adipose index (VAI), will be incorporated evaluate and compare the identifying capability of erectile dysfunction with METS-VF. This present investigation postulated that elevated METS-VF would enhance the likelihood of experiencing erectile dysfunction and outperform the other four surrogate markers of adipose accumulation in identifying erectile dysfunction.

## Methods

### Research subjects included in this study

The original material employed in this investigation is sourced from NHANES. The reliability of this database data stems from its collection through a comprehensive survey of the United States population, employing state-of-the-art and dependable technologies for data acquisition, as well as sophisticated statistical and organizational approaches for data analysis. NHANES offers comprehensive data on demographic characteristics, human examinations, disease information, and laboratory tests of each participant.

**Fig. 1:**
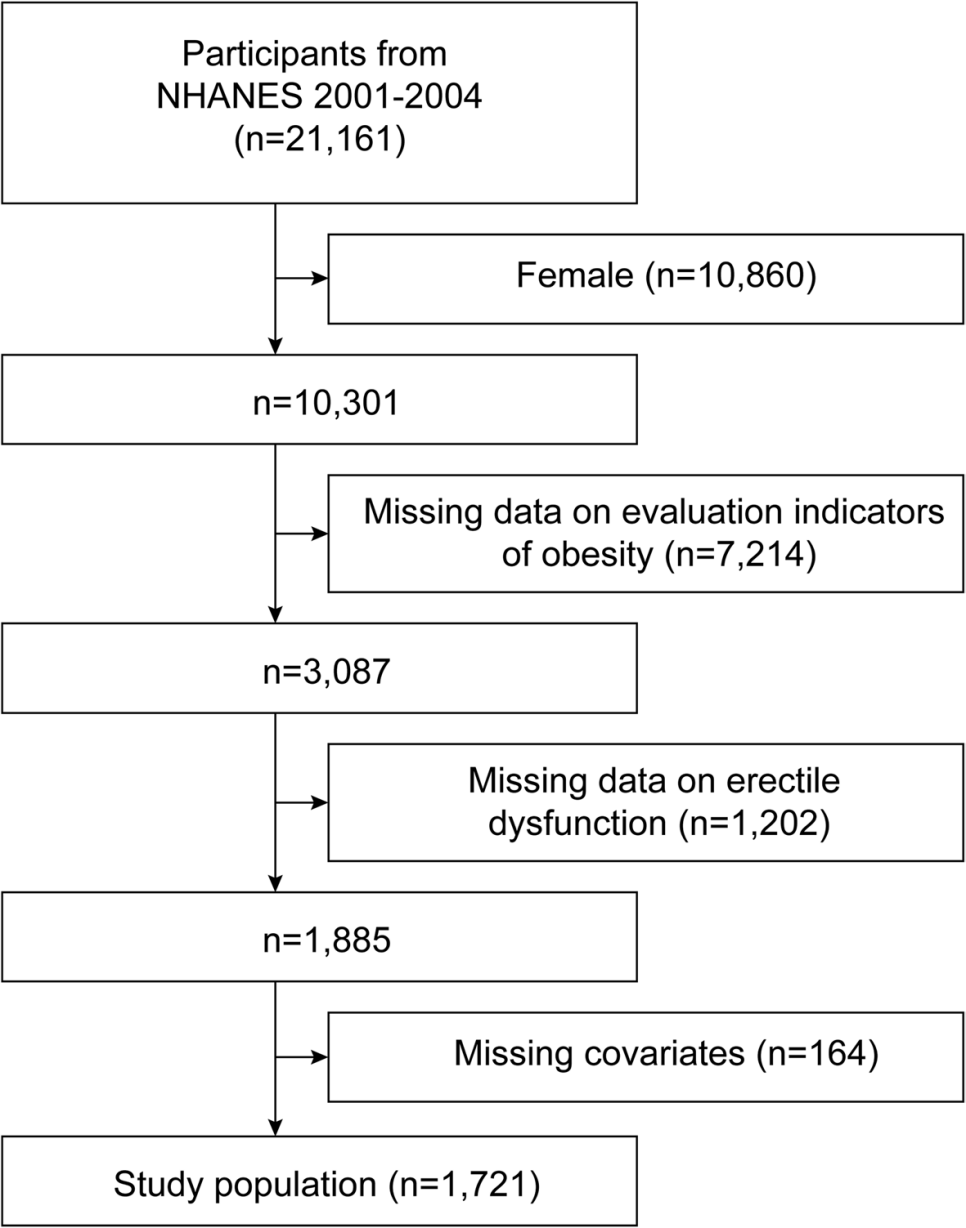
Flow chart of participants selection. NHANES, National Health and Nutrition Examination Survey

For the purpose of the current analysis, researchers selected the NHANES 2001–2004 dataset since it was the only dataset available that included information on erectile dysfunction and related variables necessary for calculating the METS-VF. Initially, a total of 21,161 subjects were enlisted for the investigation. Researchers implemented exclusion criteria for the following groups: women (*n* = 10,860), individuals without indicators of obesity (*n* = 7,214), individuals with missing erectile dysfunction data (*n* = 1,202), and individuals with missing covariate data (*n* = 164). After excluding participants who matched the aforementioned criterion, a total of 1,721 male respondents with complete data were included in the cross-sectional survey (Fig. 1). The ethical justification for the study lies in the approval granted by the National Centre for Health Statistics (NCHS) for participation in NHANES, as well as the acquisition of written informed consent from each individual.

### Definition of the novel surrogate indicators of adipose accumulation

METS-VF was considered as an exposure variable in this study. Every individual partook in a domicile interview with a physical assessment conducted at a mobile examination center (MEC). The Anthropometric Standardization Reference Manual offers comprehensive guidelines pertaining to the standardized techniques necessary for conducting anthropometric measurements [18]. Participants were instructed to abstain from consuming any food or beverages for a minimum duration of nine hours before the health test. Furthermore, researchers included WC, WHtR, LAP, and VAI as independent factors in order to evaluate their identifying efficacy in comparison to the MET-VF for the development of erectile dysfunction. The formulas pertaining to these measures are presented in the following manner. Gender was presumed to be 1 because there were no female participants in this study [19–21].

$$VAI=\frac{WC(cm)}{39.68+1.88\times BMI}\times\frac{\mathrm{triglycerides}\ (TG)(mmol/L)}{1.03}\times\frac{1.31}{\text{high}-\mathrm{density\ lipoprotein\ cholesterol}\ (\text{HDL}-\text{C})(mmol/L)}for\;males$$

$$LAP=\left(WC\left(cm\right)-65\right)\times TG\left(\frac{mmol}L\right)for\;males$$

$$WHtR=\frac{WC}{Height}$$

$$METS-IR=\frac{{\text{ln}}(2\times FPG+TG)\times BMI}{{\text{ln}}(HDL-C)}$$

 

$$METS-VF=4.466+0.01\times {\left({\text{ln}}\left({\text{METS}}-{\text{IR}}\right)\right)}^{3}+3.329\times {\left({\text{ln}}\left({\text{WHtR}}\right)\right)}^{3}+0.319\times gender+0.594\times {\text{ln}}(age)$$

### Definition of ending variables

The current study used erectile dysfunction as the ending variable. The Prostate Disease Questionnaire was employed as the principal instrument for ascertaining the presence of erectile dysfunction among participants. This questionnaire was presented via telephone interviews subsequent to the participant's completion of PSA testing and receipt of their respective outcomes. The participants were queried regarding their capacity to sustain an erection, specifically in reference to the question labeled "ability to maintain an erection (KIQ400)". If the individual expressed being "always or almost always able" or "usually able," researchers classified them as not having erectile dysfunction. Individuals who self-reported being "sometimes able" or "never able" were categorized as having erectile dysfunction.

### Measurement of mediating variables

Researchers further investigated whether homocysteine and inflammation operate as a mediation variable modulating the relationship of visceral adiposity with erectile dysfunction. Homocysteine is tested by a fully automated fluorescence polarization immunoassay. The systemic inflammation index (SII) was utilized to reflect the inflammatory status of the research individuals.

$$SII=\frac{Blood\ platelet\times Neutrophil}{Lymphocyte}$$

### Covariates

The inclusion of the following factors as covariates in this study was motivated by their potential to exert an influence on the connection between METS-VF and erectile dysfunction. The covariates encompassed in this study comprise various demographic and health-related factors, such as race, educational attainment, marital status, family income to poverty ratio, presence of hypertension and diabetes, smoking and alcohol consumption habits, levels of serum creatinine and cotinine, total cholesterol levels, history of coronary heart disease and strokes, as well as self-reported physical activity levels.

### Analytical methodology of the study

The weighted chi-square test was used to analyze the categorical data and the Mann–Whitney U test was used for continuous variables considering the non-normal distribution of continuous data. The study employed multifactor logistic regression models to examine the correlation of METS-VF with erectile dysfunction. To confirm the correctness of the analyses, three models were created. Model 1 did not involve any modifications to the variables. Model 2 was adjusted to account for the influence of race, while Model 3 was altered to account for all the covariates, including race, educational attainment, marital status, family income to poverty ratio, hypertension, diabetes, smoking, alcohol use, serum creatinine, serum cotinine, total cholesterol, coronary heart disease, strokes, and physical activity. The odds ratios (OR) and corresponding 95% confidence intervals (CI) were computed. The study employed smoothed curve fitting and threshold effects analysis to identify any nonlinear association between METS-VF and erectile dysfunction. The study examined subgroups based on educational attainment, marital status, alcohol consumption, hypertension, diabetes, coronary heart disease, stroke, physical activity, and smoking habits. Furthermore, a study was done utilizing a receiver operating characteristic (ROC) analysis to investigate the identifying efficacy of the five surrogate indicators of adipose accumulation in relation to the prevalence of erectile dysfunction. The area under the curve (AUC) of the ROC was employed as a measure of this identifying value. Then, the DeLong test realized by MedCalc software was utilized to assess the differences between the AUC values of different surrogate indicators of adipose accumulation identifying erectile dysfunction. Next, researchers applied the eXtreme Gradient Boosting (XGBoost) algorithmic model to explore the relative relevance of numerous surrogate markers of adipose accumulation for erectile dysfunction utilizing Python software. In addition to this, researchers employed multivariate logistic regression to assess the connection of METS-VF with homocysteine and inflammation. Then researchers conducted mediation analysis to determine whether homocysteine and inflammation mediated the relationship of METS-VF with erectile dysfunction. The mediated impact is equal to the indirect effect / (direct effect + indirect effect) × 100%. In the present investigation, statistical significance was defined as a *P*-value less than 0.05. The primary statistical tool utilized for the analyses in this investigation was R version 3.4.3, developed by The R Foundation. Additionally, the Empower program and MedCalc software were also employed for the analysis of this study. The analysis of mediating effects was performed through the “PROCESS” software package of SPSS.

## Results of data analysis for the study

### Demographic features of the participants in the study

This study had a cohort of 1,721 male subjects, with an average age of 49.4 ± 18.1 years (Table 1). Among these individuals, 1,233 did not exhibit symptoms of erectile dysfunction, while 488 experienced erectile dysfunction. The data reveals that individuals who identify as non-Hispanic whites constitute the most significant segment of the erectile dysfunction population. Furthermore, individuals with erectile dysfunction exhibited advanced age, a greater incidence of hypertension, diabetes, stroke, coronary heart disease, and a higher propensity for smoking. However, they displayed lower socioeconomic status, educational attainment, and physical activity levels. Furthermore, it was shown that the group with erectile dysfunction exhibited higher values for all five surrogate indicators of adipose accumulation in comparison to the subjects without erectile dysfunction. Surprisingly, no individuals with erectile dysfunction were found to have a significantly higher BMI than the non-erectile dysfunction group.

**Table 1:** Baseline characteristics of the study population stratified by presence or absence of erectile dysfunction

	**Total (** ***n*** ** = 1,721)**	**No erectile dysfunction (** ***n*** ** = 1,233)**	**Erectile dysfunction** **(** ***n*** ** = 488)**	***P*** **-value**
**Age (years)**	49.4 ± 18.1	43.3 ± 15.3	64.9 ± 15.0	< 0.001
**Family income to poverty ratio** **BMI (kg/m^2)**	2.86 ± 1.60 28.05 ± 5.35	2.93 ± 1.62 27.88 ± 5.16	2.68 ± 1.56 28.49 ± 5.79	0.005 0.089
**Serum Cotinine (ng/mL)**	68.72 ± 133.16	76.65 ± 141.16	48.68 ± 107.95	< 0.001
**Total cholesterol (mg/dL)**	198.61 ± 43.38	199.46 ± 42.27	196.45 ± 46.02	0.110
**Serum Creatinine(mg/dL)**	1.03 ± 0.43	1.01 ± 0.40	1.10 ± 0.49	< 0.001
**Race**				0.003
Mexican American	360 (20.92%)	256 (20.76%)	104 (21.31%)	
Other Hispanic	52 (3.02%)	34 (2.76%)	18 (3.69%)	
Non-Hispanic White	956 (55.55%)	661 (53.61%)	295 (60.45%)	
Non-Hispanic Black	307 (17.84%)	244 (19.79%)	63 (12.91%)	
Other Race	46 (2.67%)	38 (3.08%)	8 (1.64%)	
**Education level**				< 0.001
Less than high school	474 (27.54%)	284 (23.03%)	190 (38.93%)	
High school	430 (24.99%)	332 (26.93%)	98 (20.08%)	
More than high school	817 (47.47%)	617 (50.04%)	200 (40.98%)	
**Marital status**				< 0.001
Married/Living with partners	1198 (69.61%)	828 (67.15%)	370 (75.82%)	
Widowed/Divorced/Separated	224 (13.02%)	135 (10.95%)	89 (18.24%)	
Never married	299 (17.37%)	270 (21.90%)	29 (5.94%)	
**Alcohol use**				0.082
Yes	1429 (83.03%)	1036 (84.02%)	393 (80.53%)	
No	292 (16.97%)	197 (15.98%)	95 (19.47%)	
**Hypertension**				< 0.001
Yes	533 (30.97%)	279 (22.63%)	254 (52.05%)	
No	1188 (69.03%)	954 (77.37%)	234 (47.95%)	
**Diabetes**				< 0.001
Yes	183 (10.63%)	63 (5.11%)	120 (24.59%)	
No	1538 (89.37%)	1170 (94.89%)	368 (75.41%)	
**Coronary heart disease**				< 0.001
Yes	116 (6.74%)	41 (3.33%)	75 (15.37%)	
No	1605 (93.26%)	1192 (96.67%)	413 (84.63%)	
**Strokes**				< 0.001
Yes	62 (3.60%)	15 (1.22%)	47 (9.63%)	
No	1659 (96.40%)	1218 (98.78%)	441 (90.37%)	
**Physical activity**				< 0.001
Yes	1065 (61.88%)	797 (64.64%)	268 (54.92%)	
No	656 (38.12%)	436 (35.36%)	220 (45.08%)	
**Smoking**				< 0.001
Yes	1027 (59.67%)	673 (54.58%)	354 (72.54%)	
No	694 (40.33%)	560 (45.42%)	134 (27.46%)	
**Surrogate indicators of adipose accumulation**				
** METS-VF**	6.86 ± 0.71	6.72 ± 0.72	7.23 ± 0.53	< 0.001
** WC**	100.06 ± 14.33	98.51 ± 13.94	103.99 ± 14.56	< 0.001
** LAP**	70.61 ± 95.49	66.01 ± 90.95	82.21 ± 105.30	< 0.001
** VAI**	2.48 ± 3.34	2.41 ± 3.43	2.67 ± 3.09	<0.001
** WHtR**	0.57 ± 0.08	0.56 ± 0.08	0.60 ± 0.08	< 0.001

*BMI* body mass index, *METS-VF* metabolism score for visceral fat, *WC* waist circumference, *LAP* lipid accumulation product, *VAI* visceral adiposity index, *WHtR* waist-to-height ratio

### Higher METS-VF is associated with erectile dysfunction

Table 2 presents the correlation between METS-VF and erectile dysfunction. After adjusting for all covariates of the investigation, it was observed that each unit increase in METS-VF was associated with a 141% increase in the prevalence of erectile dysfunction (OR = 2.41, 95% CI: 1.86–3.11, *P* < 0.0001). The METS-VF variable underwent a transformation from a continuous to a categorical form (tertiles). In the model that was fully adjusted, it was found that the occurrence of erectile dysfunction in the highest METS-VF tertile was 3.21 times more than in the lowest METS-VF tertile (OR = 3.21, 95% CI: 2.24–4.59, *P* < 0.0001). Similarly, similar results were observed in Model 1 and Model 2. Furthermore, it is worth noting that all *P*-trends exhibited statistical significance.

**Table 2:** Analyzing the association between METS-VF and erectile dysfunction using multivariate logistic regression

Exposure	OR (95% CI), *P*-value		
	**Model 1**	**Model 2**	**Model 3**
METS-VF	4.48 (3.57, 5.63) < 0.0001	4.45 (3.53, 5.61) < 0.0001	2.41 (1.86, 3.11) < 0.0001
METS-VF tertiles			
T1 (3.87–6.70)	1.00 (Reference)	1.00 (Reference)	1.00 (Reference)
T2 (6.71–7.24)	2.38 (1.73, 3.27) < 0.0001	2.33 (1.69, 3.22) < 0.0001	1.61 (1.12, 2.31) 0.0095
T3 (7.25–8.10)	7.41 (5.47, 10.03) < 0.0001	7.20 (5.30, 9.79) < 0.0001	3.21 (2.24, 4.59) < 0.0001
*p*-trend	< 0.0001	< 0.0001	< 0.0001

Model 1, crude model;

Model 2, adjusted for race;

Model 3, adjusted for race, education level, marital status, family income to poverty ratio, hypertension, diabetes, smoking, alcohol use, serum creatinine, serum cotinine, total cholesterol, coronary heart disease, strokes, and physical activity

*METS-VF* metabolism score for visceral fat, *OR* odds ratio, *CI* Confidence interval

**Fig. 2:**
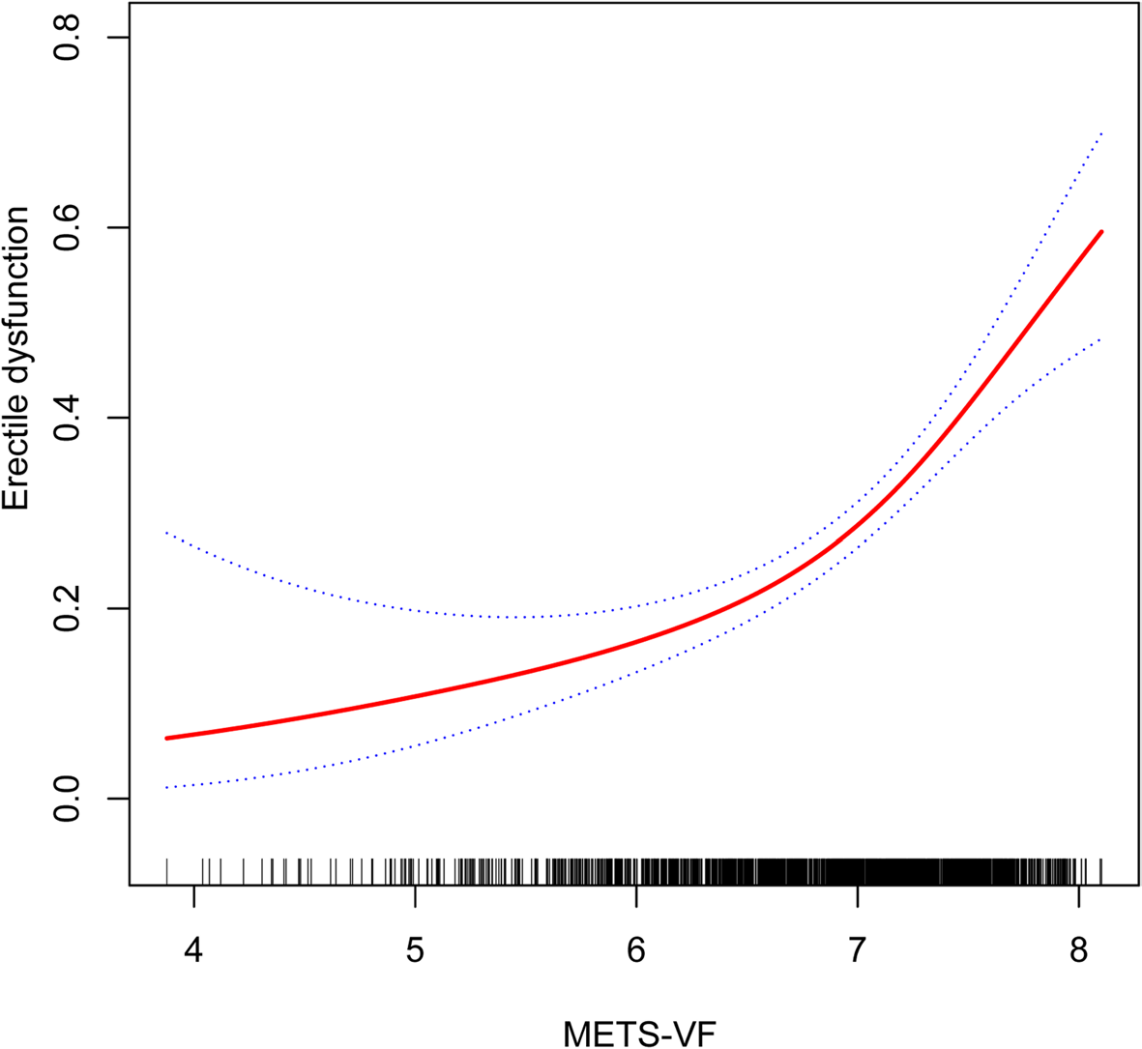
Association between METS-VF and erectile dysfunction. Adjustments have been made for all covariates in the investigation. METS-VF, metabolism score for visceral fat

Figure 2 and Table 3 show the results of the smoothed curve fitting and threshold effects analysis. After adjusting for all considered covariates, a J-shaped relationship was found of METS-VF with erectile dysfunction, with a breakpoint of 7.01. This means that there is a notable change in the relationship of METS-VF with erectile dysfunction at this specific value. Specifically, when METS-VF was less than 7.01, the occurrence of sexual disorder onset increased slowly with METS-VF, and the occurrence of sexual disorder onset increased by 62% for every 1 unit increase in METS-VF (OR = 1.62, 95% CI: 1.10–2.38, *P* = 0.0141). Whereas, when METS-VF was greater than 7.01, the prevalence of erectile dysfunction increased rapidly with increasing METS-VF, with a 337% increase in the prevalence of erectile dysfunction for every 1 unit increase in METS-VF (OR = 4.37, 95% CI: 2.55–7.50, *P* = 0.0141). This relationship was supported by a log-likelihood ratio test with a *P*-value of 0.013, as shown in Table 3.

**Table 3:** Results of threshold effects analysis

Ending variables	Erectile dysfunction
	**Fitting by standard linear model**
**OR (95% CI) ** ***P*** **-value**	2.41 (1.86, 3.11) < 0.0001
	**Fitting by two-piecewise linear model**
**Breakpoint (K)**	7.01
**OR1 (< K)**	1.62 (1.10, 2.38) 0.0141
**OR1 (> K)**	4.37 (2.55, 7.50) < 0.0001
**Log likelihood ratio**	0.013

*OR* odds ratio, *CI* Confidence interval

### Results of subgroup analysis

**Fig. 3:**
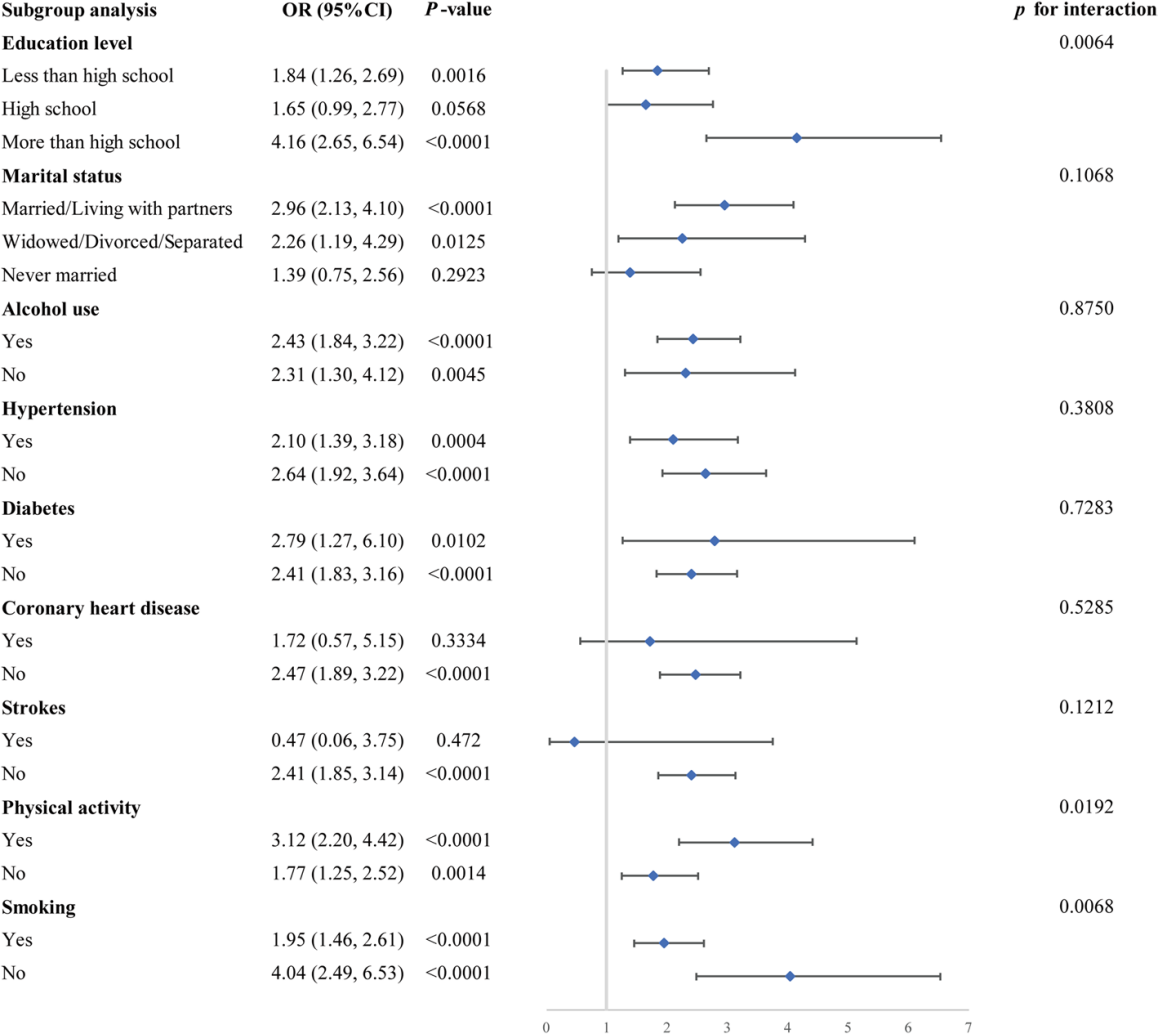
Results of subgroup analysis. Adjustments have been made for all covariates in the investigation. OR, odds ratio; CI, Confidence interval

For the purpose of evaluating the robustness of the relationship of METS-VF with erectile dysfunction in different populations, researchers tested for interactions between education, marital status, alcohol use, physical exercise, smoking, and previous medical conditions including hypertension, diabetes, coronary artery disease, and stroke. However, as shown in Fig. 3, this association between the indicator of visceral obesity and erectile dysfunction was not consistent across subgroups after adjusting all confounders. Significant interactions were observed in the subgroups of educational level, physical activity, and smoking. Those with education below and above high school showed a favorable relationship of METS-VF with erectile dysfunction, but a statistically significant relationship of METS-VF with erectile dysfunction was not found in those with high school education. Additionally, although a positive relationship of METS-VF with erectile dysfunction was present in subjects with and without physical activity, a more significant association could be found in populations with physical activity. Similarly, in the subgroup of individuals who smoke, there was a greater association of METS-VF with erectile dysfunction in those who do not smoke, in comparison to those who do smoke. In contrast, no statistically significant *P* for interaction was discovered in subgroups of marital status, alcohol use, hypertension, diabetes, coronary heart disease, and stroke.

### METS-VF may be a better indicator of identifying erectile dysfunction

**Fig. 4:**
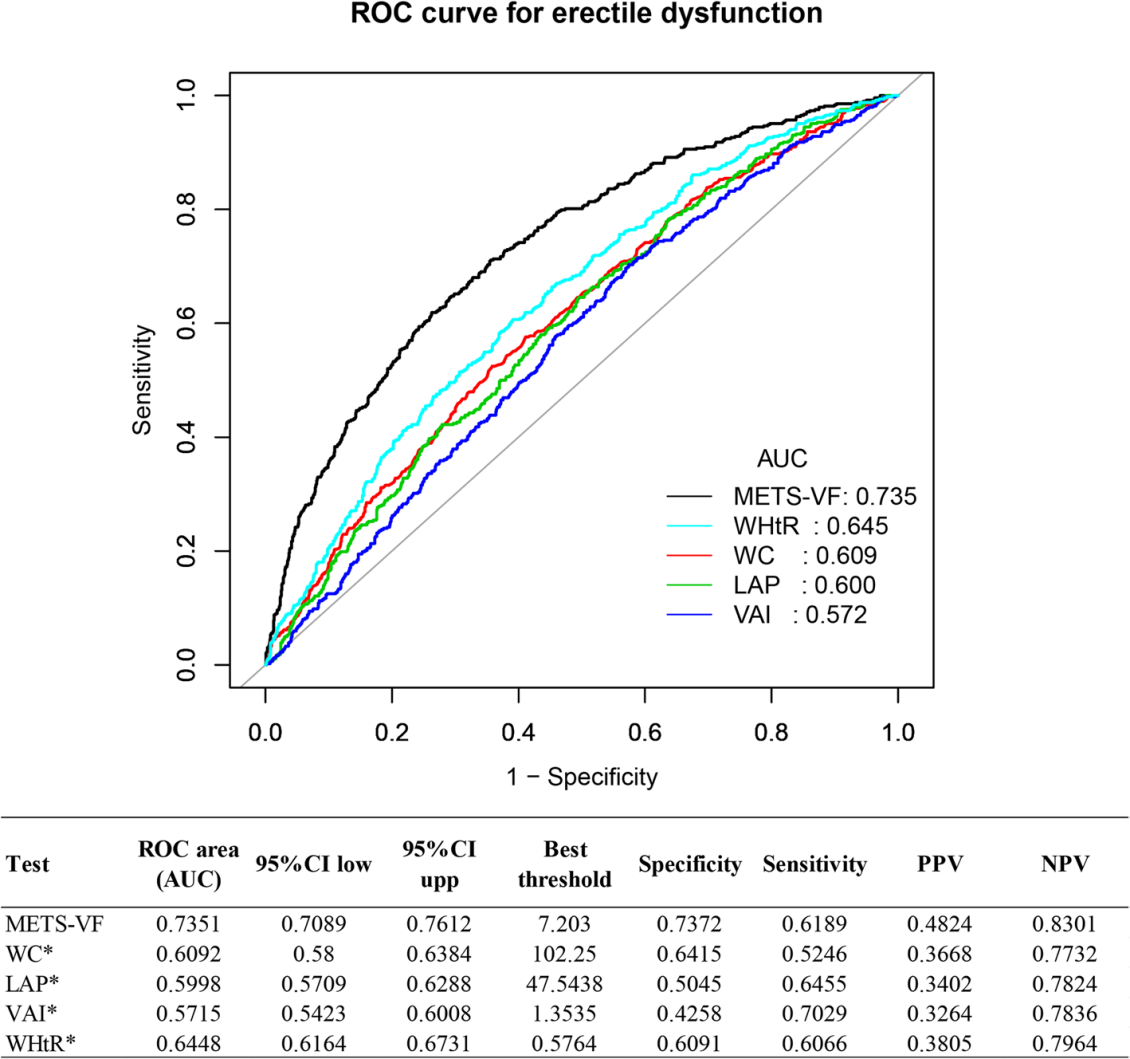
Comparison of the predictive power for evaluation indicators of obesity. *When the AUC value of this indicator was compared to the AUC value of the METS-VF the result obtained was *P* < 0.05. *METS-VF* metabolism score for visceral fat, *WC* waist circumference, *LAP* lipid accumulation product, *VAI* visceral adiposity index, *WHtR* waist-to-height ratio, *PPV* positive predictive value, *NPV* negative predictive value

Figure 4 presents the AUC values for the five surrogate indicators of adipose accumulation employed in the identification of erectile dysfunction. All five surrogate indicators demonstrated efficacy in predicting erectile dysfunction. The outcome discovered that the METS-VF metric exhibited the greatest AUC value of 0.7351 (95% CI: 0.7089–0.7612). This value surpassed the AUC values of other metrics such as WC, LAP, VAI, and WHtR. The METS-VF demonstrated an optimum cutoff with a value of 7.2030 (Specificity = 0.7372, Sensitivity = 0.6189).

## Results of the XGBoost model

**Fig. 5:**
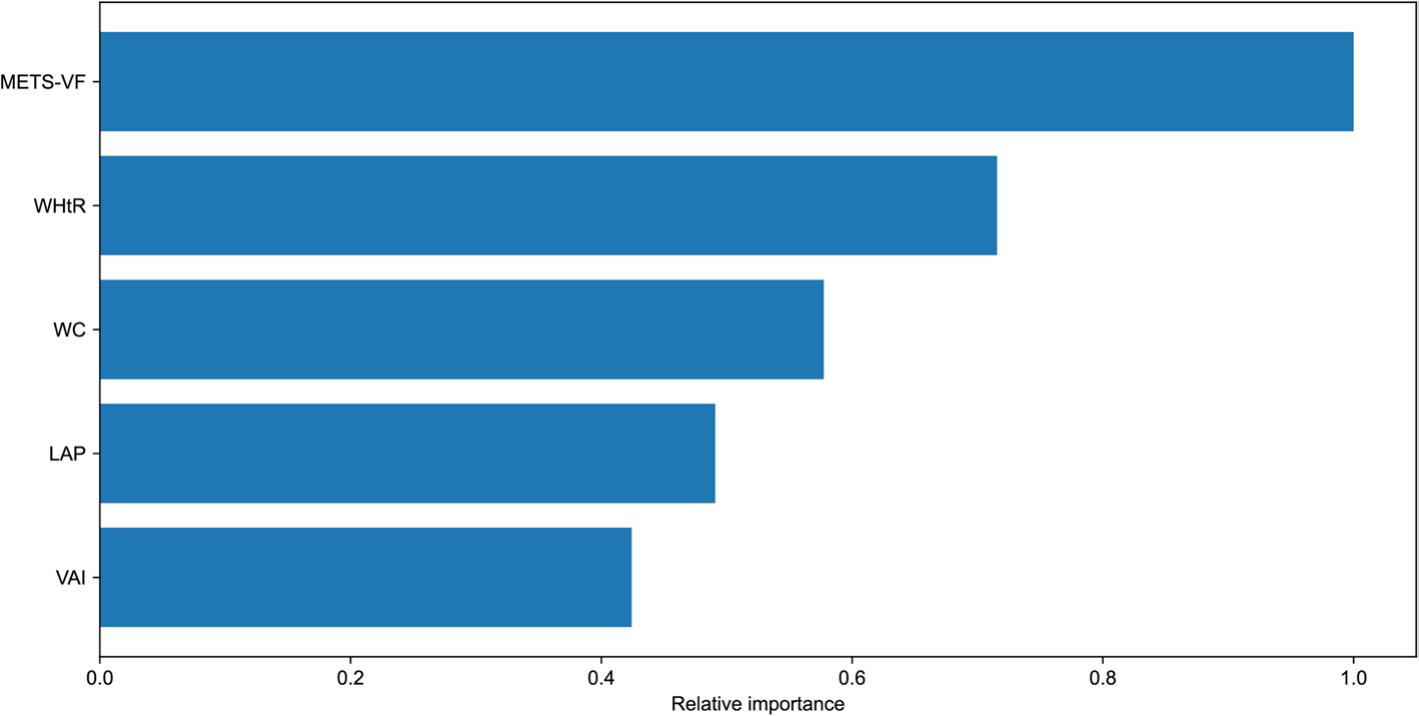
Using XGBoost modeling to assess the relative importance of surrogate indicators of adipose accumulation for erectile dysfunction. *METS-VF* metabolism score for visceral fat, *WC* waist circumference, *LAP* lipid accumulation product, *VAI* visceral adiposity index, *WHtR* waist-to-height ratio

The XGBoost model is an emerging machine-learning approach that researchers employed to identify the relative significance of multiple surrogate indicators of adipose accumulation for erectile dysfunction. The results in Fig. 5 revealed that METS-VF is the most critical sign of erectile dysfunction among American men, followed by WHtR, WC, LAP, and VAI in order of relative relevance.

**Fig. 6:**
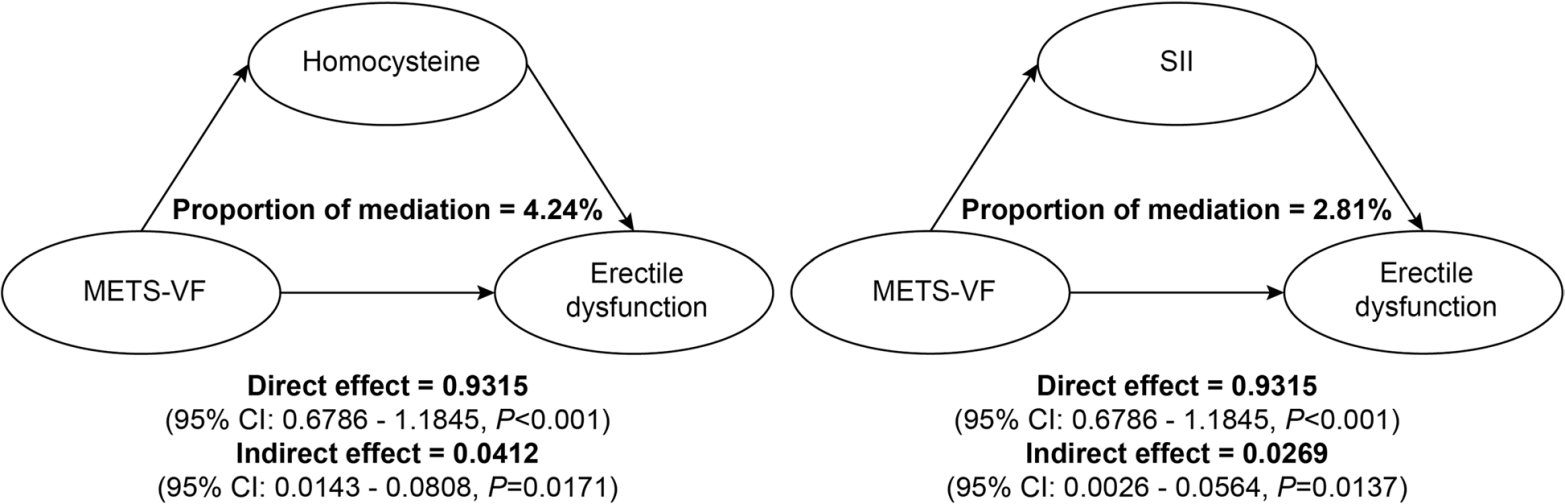
Results of the mediation effects analysis. *METS-VF* metabolism score for visceral fat, *SII* systemic inflammation index, *CI* Confidence interval

### Homocysteine and inflammation mediate the relationship of METS-VF with erectile dysfunction

Table 4 displays the outcomes of the multiple logistic regression analysis of METS-VF with homocysteine and inflammation. After accounting for all factors, the investigation found a clear correlation of METS-VF with both homocysteine levels and inflammation.n. Researchers then conducted mediation analyses based on this foundation. The mediation analysis results indicated that the mediation rates for homocysteine and SII were 4.24% and 2.81%, respectively (Fig. 6).

**Table 4:** Analyzing the association of METS-VF with homocysteine and SII using multivariate logistic regression

METS-VF	β (95%CI), *P*-value		
	**Model 1**	**Model 2**	**Model 3**
Homocysteine	0.95 (0.68, 1.22) < 0.0001	1.02 (0.74, 1.29) < 0.0001	0.48 (0.18, 0.78) 0.0016
SII	55.58 (27.62, 83.53) 0.0001	41.16 (12.55, 69.77) 0.0049	49.15 (15.31, 82.99) 0.0045

Model 1, crude model;

Model 2, adjusted for race;

Model 3, adjusted for race, education level, marital status, family income to poverty ratio, hypertension, diabetes, smoking, alcohol use, serum creatinine, serum cotinine, total cholesterol, coronary heart disease, strokes, and physical activity

*OR* odds ratio, *CI* Confidence interval, *METS-VF* metabolism score for visceral fat, *SII* systemic inflammation index

## Discussion

The study included a sample size of 1,721 male participants. The current study shows that there is a significant favorable relationship of METS-VF with erectile dysfunction and that the relationship between these two variables is J-shaped. Additionally, the subgroup analysis findings suggest that the relationship of METS-VF with erectile dysfunction may be modified by education level, physical activity, and smoking.

In contemporary society, obesity is being recognized as a vital health concern that demands attention, particularly in relation to male reproductive health. The most often used anthropometric indicator of obesity is BMI and research on the connection between obesity and male reproductive function has been done in the past. Prospective studies have shown that obesity is positively associated with a higher probability of erectile dysfunction [22, 23]. Whereas, researchers note that the primary focus of these investigations predominantly employed the BMI as a means to evaluate obesity, a measure widely recognized for its effectiveness in detecting overall obesity but limited in its ability to accurately analyze the distribution of adipose tissue [11]. Not all adipose tissue is deleterious; the presence of fat in the thigh region may potentially mitigate the likelihood of developing cardiovascular disease [24]. When an individual's BMI falls within the normal range, the presence of central obesity can be indicative of an increased hazard to several illness conditions [25, 26]. Yassin et al. discovered that WC was a better predictor of sexual symptoms related to erectile dysfunction compared to BMI [27]. Additionally, an investigation performed by Lin et al. discovered that there is no statistically noteworthy relationship between BMI and erectile dysfunction [28]. Another study from Canada found that a high WC is linked to a higher likelihood of experiencing erectile dysfunction, although BMI is not [29]. Similarly, a meta-analysis comprising 45 investigations did not find an association between BMI in the overweight range and erectile dysfunction [30]. Therefore, for more rigorous considerations, the use of a central lipid accumulation index is a better choice for the assessment of erectile dysfunction.

Romero-Corral et al. conducted a randomized controlled trial which found that in young individuals of normal weight, an accumulation of visceral fat, but not subcutaneous fat, results in endothelial dysfunction, which is a crucial factor in erectile dysfunction [31]. It is crucial to emphasise that the researchers in this research utilized dual-energy X-rays to evaluate visceral fat. Despite the high accuracy of imaging counts to assess visceral fat, they are not suitable for widespread use in epidemiologic studies or routine clinical testing because of the high operational requirements and cost. Consequently, the development of straightforward and trustworthy indicators for the evaluation of visceral obesity is crucial. Central obesity has been found to be an independent predictor of erectile dysfunction [12]. While WC is frequently employed as a measure to evaluate central obesity, it is worth noting that persons with comparable WC values may have an overestimation of risk if they are taller, and conversely, an underestimation of risk if they are shorter [32]. Consequently, to overcome the shortcomings of WC, a number of innovative indicators based on WC have been suggested to evaluate central obesity. Previous research has investigated the association between these novel indicators and the occurrence of erectile dysfunction. VAI is commonly employed in epidemiological research as a significant measure of visceral adiposity and insulin sensitivity [33]. Akdemir AO et al. showed that compared to healthy individuals, erectile dysfunction patients have a substantially higher VAI [34]. Xu et al. verified that an increased VAI is a standalone predictor of erectile dysfunction through the use of a larger sample size [35]. In addition, LAP, a strong indicator of central adipose accumulation, is independently correlated with androgen deficiency and low serum total testosterone levels [36, 37]. The current research offers innovative perspectives on searching for novel surrogate indicators of adipose accumulation, METS-VF, in connection to erectile dysfunction. Specifically, for every one-unit rise in METS-VF, the occurrance of erectile dysfunction increased by 141%. More notably, this study found that the relationship of METS-VF with erectile dysfunction was a J-shaped nonlinear relationship. METS-VF showed a favorable relationship with erectile dysfunction when the value of METS-VF was higher than 7.01. Researchers hypothesize that when lipid accumulation reaches a certain level, various metabolic disorders may have a synergistic effect and seriously jeopardize the reproductive health of patients. The blow of this harm to the sexual health of the patient is enormous, that is the reason why patients should strive to maintain a low level of METS-VF.

Additionally, the findings from ROC studies indicate that the METS-VF exhibits a higher level of identifying capability for erectile dysfunction in comparison to the four remaining evaluation indicators of abdominal obesity. The XGBoost model results verified that METS-VF is a more significant indication of erectile dysfunction. The three primary components of the METS-VF are the demographic indicators of age and sex, the surrogate indicators of adipose accumulation WHtR, and the insulin resistance evaluation indication METS-IR. Undoubtedly, the likelihood of developing erectile dysfunction rises with age, and there is evidence to suggest that aging is a significant factor in visceral fat accumulation [38]. Additionally, METS-IR has an excellent degree of accuracy (AUC = 0.84) in identifying insulin resistance and was confirmed using a glucose-hyperinsulinemia clamp, a gold standard for assessing insulin resistance [39]. Reduced vascular nitric oxide (NO) generation and decreased insulin-induced vasodilation are symbols of insulin resistance, and both conditions might result in vascular endothelial dysfunction, a significant pathophysiological process that underlies erectile dysfunction [40]. These two advantages are not shared by the other four human evaluation indicators of obesity markers. Additionally, it's also important to note that lipid biochemical indicators included in METS-IR show both the quality and functional state of body fat. Moreover, the METS-VF calculation includes the WHtR. The current research of the ROC curve and XGBoost algorithm revealed that for erectile dysfunction, WHtR is the second most significant parameter following METS-VF. For the above reasons, METS-VF demonstrates more powerful capability in evaluating erectile dysfunction. Also, with an AUC of 0.78, dual-energy X-ray validation of METS-VF's high accuracy in visceral fat accumulation demonstrates its superiority over BMI, WC, weight, and WHtR [41]. Another Turkish study also emphasized that METS-VF has the highest predictive value in identifying visceral fat, significantly higher than LAP and VAI [42]. Furthermore, it should be noted that the VAI and LAP were not intended to be used in evaluating visceral fat [38]. The findings of current investigation also indicated that, out of the five measures of central obesity, the VAI and LAP had the lowest predictive performance for erectile dysfunction. These findings suggest that the VAI and LAP are less useful than METS-VF in identifying diseases linked to the accumulation of visceral obesity. Considering the calculation of METS-VF is derived from commonly used clinical indicators, as a result, even in situations where costs are limited, METS-VF can still be considered the preferred indicator to assess visceral fat. It is also important to highlight that it can effectively reflect multiple risk factors and pathophysiological mechanisms of erectile dysfunction. In conclusion, the study's findings offer fresh proof in support of the theory that central obesity raises the prevalence of erectile dysfunction.

Despite being a relatively new indicator that was first proposed in 2020, the METS-VF offers outstanding assessment capabilities across a wide range of disorders. In their study, Yu et al. discovered that the METS-VF had a robust predictive capacity for chronic kidney disease (CKD) when in comparison to alternative evaluation markers of central adiposity [43]. Additionally, in comparison to other evaluation indicators of obesity, METS-VF demonstrates reliability and applicability as a predictor of hypertension and diabetes within the Chinese population [44, 45]. A 36,876-person study found a favorable correlation between METS-VF and asthma [21]. For non-obese women, the METS-VF is helpful in directing the prevention and management of hyperuricemia [46]. The close association of these diseases with erectile dysfunction has been confirmed by many studies [47–51]. These offer indirect proof of the robust identifying ability of METS-VF for erectile dysfunction. It is acceptable to assume that the METS-VF is useful in identifying the increasing prevalence of erectile dysfunction based on prior studies as well as the findings of the current investigation.

The exact biological processes through which visceral obesity causes erectile dysfunction are not completely understood. The current research suggests that visceral obesity may elevate the likelihood of erectile dysfunction in the population by raising homocysteine levels in the blood via mediated effects analysis. A meta-analysis of 14 research validated that homocysteine levels were elevated in obese populations, aligning witht the results of current research [52]. Obese patients are at risk of insulin resistance, leading to increased insulin secretion to regulate blood glucose levels. This excess insulin can elevate circulating homocysteine concentrations by inhibiting hepatic cystathionine-B-synthase activity on the one hand, and on the other hand, insulin inhibits homocysteine catabolic conversion [53, 54]. Elevated homocysteine levels in the body hinder the production of NO in the corpus cavernosum [55]. NO is crucial for regulating endothelial function. It stimulates the production of cyclic guanosine sapogenins by activating soluble guanylate cyclase, leading to elevated intracorporeal pressure, vasodilation, and erection [56]. In addition, Elevated levels of homocysteine can hinder the activation and expression of Endothelial Nitric Oxide Synthase (eNOS), promote eNOS uncoupling, and boost the production of oxygen free radicals, resulting in endothelial dysfunction [57]. Furthermore, this study discovered that inflammation also mediated the relationship between MET-VF and erectile dysfunction. Inflammatory substances such as TNF-α suppress the eNOS gene expression in endothelial cells, causing endothelial dysfunction and subsequently leading to erectile dysfunction [58, 59].

Meanwhile, it is important to highlight that subgroup analysis revealed the correlation of METS-VF with erectile dysfunction may be modified by education level, physical activity, and smoking. Therefore, researchers attempted to further clarify the reasons for the emergence of the results of the subgroup analyses. Prior research has demonstrated that individuals with lower levels of education and a sedentary lifestyle are more susceptible to experiencing erectile dysfunction [60, 61]. Additionally, it has been found that smoking elevates the probability of developing moderate or severe erectile dysfunction twofold [62]. On the other hand, physical activity has been shown to exert a beneficial influence on the reduction of visceral accumulation and the promotion of testosterone production [63]. Moreover, according to a study conducted in Finland, there is evidence to suggest that those with higher levels of education may experience a decreased likelihood of developing erectile dysfunction [64]. Lower levels of education, smoking, and lack of exercise are indeed considered risk factors for erectile dysfunction, and they may exert a positive influence on increasing the likelihood of developing erectile dysfunction compared to obesity in this population. Therefore, the influence of obesity on the probability of experiencing erectile dysfunction may be relatively limited in these populations. On the other hand, individuals who are more educated, non-smokers, and physically active are regarded as a healthier population and no other potential risk factors exist, the correlation between obesity and erectile dysfunction is expected to be more pronounced.

### Study strengths and limitations

The current study possesses some notable strengths that deserve recognition. This work represents the first insight into the correlation of METS-VF with erectile dysfunction and compares its predictive performance of erectile dysfunction with several evaluation indicators of obesity. Furthermore, researchers accounted for potential confounding factors in the multiple logistic regression analyses. Additionally, subgroup analyses were used to explore different population settings in this correlation. Nevertheless, it is crucial to recognize that the present investigation does have many limitations. The current study employed a cross-sectional design, which precludes the establishment of a causal link between METS-VF and erectile dysfunction. Furthermore, it is important to consider the potential presence of recall bias in the assessment of erectile dysfunction, as it mostly relies on the use of questionnaires. Furthermore, given that this study was done exclusively on a sample from the United States, it is imperative to undertake more extensive investigations in order to ascertain the generalizability of the findings to populations in different geographical regions.

## Conclusion

The study findings indicated a favorable correlation of METS-VF with erectile dysfunction. Furthermore, METS-VF demonstrated a higher identifying accuracy for erectile dysfunction when compared to the other four surrogate markers of adipose accumulation. The current investigation offers novel perspectives on the impact of lipid metabolism on erectile dysfunction. The affordability and accessibility of METS-VF make it a valuable marker in various facets of the condition. Hence, it is highly advisable to incorporate METS-VF as a standard indicator of the effectiveness of clinical treatments for erectile dysfunction. Simultaneously, the significance of METS-VF in the prevention of erectile dysfunction should not be overlooked. Medical professionals and caregivers are expected to decrease METS-VF by making changes to the daily diet and exercise routines of individuals who are at risk. This can help lower the chances of developing erectile dysfunction and ultimately support the overall reproductive health of men.

## Data Availability

The original data were retrieved from https://wwwn.cdc.gov/nchs/nhanes/Default.aspx.
